# Thoracoscopic Massive Bleeding After Firing Mediastinal Trunk of the Pulmonary Artery With Calcified Lymph Node

**DOI:** 10.1111/1759-7714.70092

**Published:** 2025-05-22

**Authors:** Alfonso Fiorelli, Beatrice Leonardi, Maria Marvulli, Francesca Capasso, Vincenzo Di Filippo, Francesco Coppolino, Giovanni Vicidomini

**Affiliations:** ^1^ Thoracic Surgery Unit Università della Campania “Luigi Vanvitelli” Caserta Italy; ^2^ Anesthesiology Unit Università della Campania “Luigi Vanvitelli” Caserta Italy

**Keywords:** calcified lymph node, lung cancer, thoracoscopic bleeding

## Abstract

The thoracoscopic management of hilar calcified lymph nodes is a technical challenge as the dense adhesions with the bronchus and vessels prevented a safe dissection. Herein, we reported the unexpected bleeding after firing the mediastinal trunk of the pulmonary artery with calcified lymph nodes during the completion of thoracoscopic right upper lobectomy for the management of lung cancer. The bleeding was successfully fixed by an emergent thoracotomy. We used a standard white vascular cartridge that probably was unable to staple a thick tissue, such as the vessel with calcified lymph node. Thus, the best strategy remained to cut the pulmonary artery where the lymph nodes were not attached, and the plasty of the pulmonary artery should be considered if the lymph nodes could not be dissected from the vessels. If the surgeons were not confident to manage this situation under thoracoscopy, conversion to thoracotomy should never be forgotten. Open surgery could facilitate the dissection of calcified lymph nodes and safely fix unexpected bleeding due to vascular lesions.

## Graphical abstract

CT and intraoperative view showed the presence of a hilar lymph node. Unexpected massive bleeding occurred after firing the mediastinal trunk with the calcified lymph node. The complication was successfully faced by emergent thoracotomy.

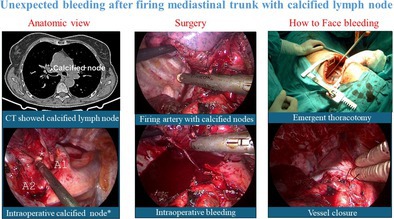

## 1 Introduction

The thoracoscopic management of hilar calcified lymph nodes is a technical challenge as the dense adhesions with the bronchus and vessels prevented a safe dissection [1, 2, 3].

Herein, we reported the unexpected bleeding after stapling the mediastinal trunk of the pulmonary artery for the presence of hilar lymph nodes during the completion of thoracoscopy right upper lobectomy. An emergent thoracotomy was needed to fix the bleeding.

## 2 Case Report

**FIGURE 1:**
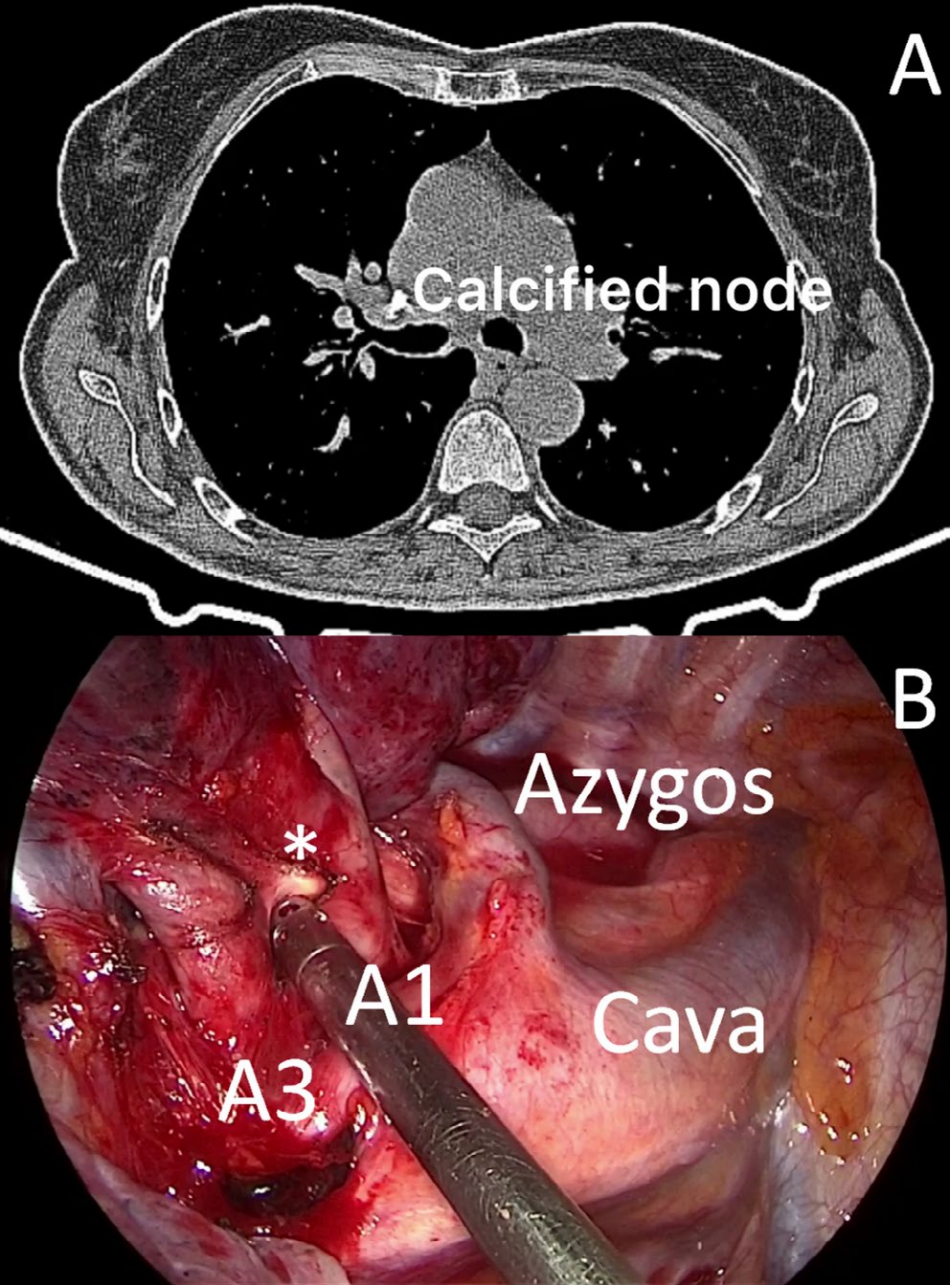
Chest CT scan (A) and intraoperative view (B) showed the presence of a calcified lymph node (*) between the A1 and the A3 branches of the mediastinal trunk of the pulmonary artery.

A 67‐year old woman was referred to our hospital for management of adenocarcinoma of the right upper lobe (T1bN0M0). Whole‐body CT scan showed a 20 mm nodule within the right upper lobe and the presence of calcified lymph nodes near the mediastinal trunk of the PA (Figure 1A). The nodule presented pathological uptake on the PET scan and no other lesions were found. FNAB CT‐guided diagnosed adenocarcinoma. Cardio‐respiratory function was within normal; thus, the patient was scheduled for thoracoscopic lobectomy.

**FIGURE 2:**
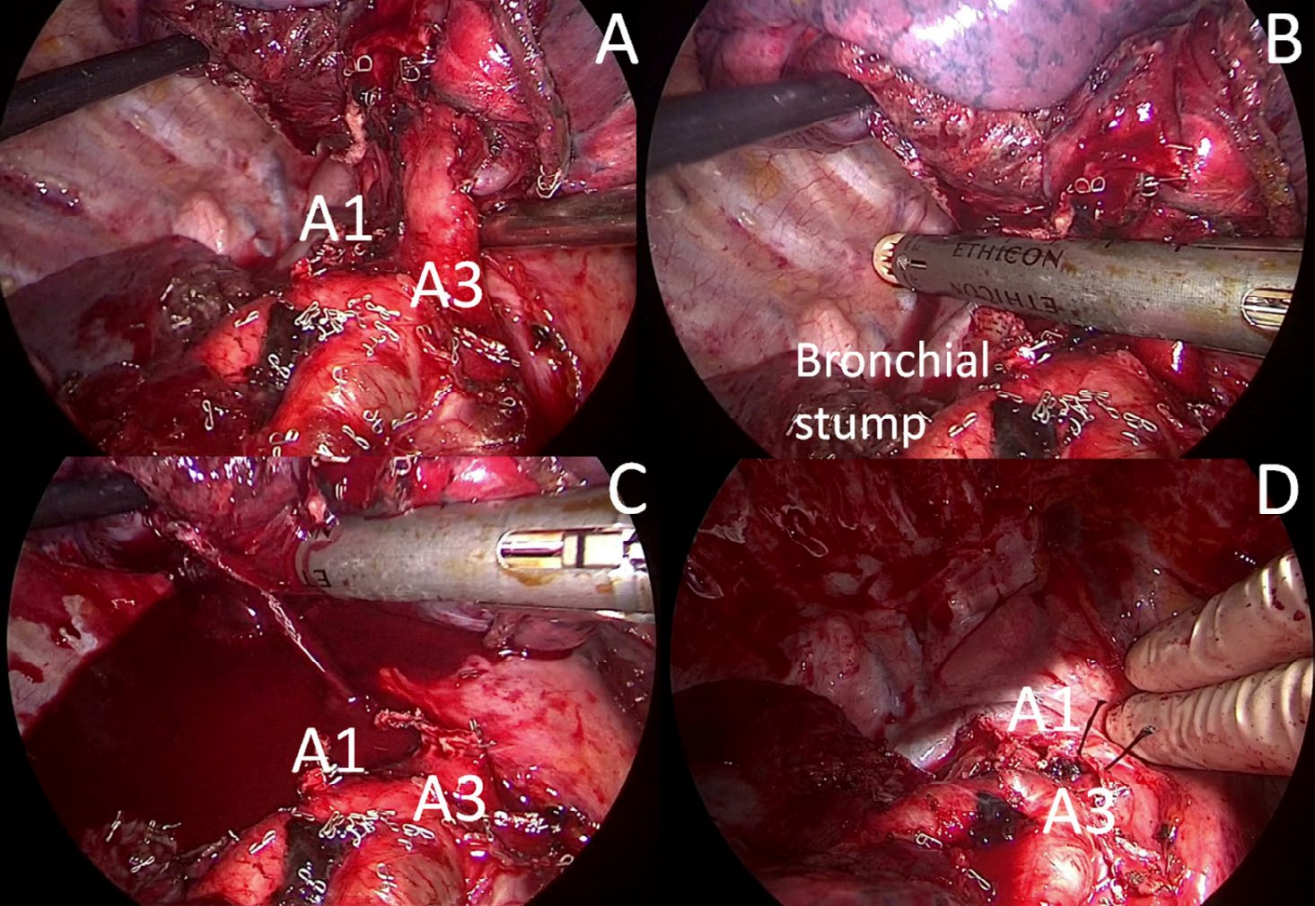
The mediastinal trunk was isolated (A) and stapled together with the calcified lymph node (B). After opening the staple jaws, massive bleeding from the A1 branch occurred (C). The A1 trunk was ligated with resolution of the bleeding (D).

A standardized three‐port anterior approach was performed. Pulmonary vein, horizontal fissure, and oblique fissure between S2–S6 segment; A2 branch of PA and right upper bronchus were sequentially isolated and stapled. Finally, we approached the mediastinal trunk of PA. The dissection of the calcified lymph node stretched the vessels with a high risk of injury (Figure 1B). Thus, the mediastinal trunk was stapled together with the lymph node using a 60 white vascular cartridge (Figure 2A). When the staple jaws were opened, there was unexpected bleeding from the A1 branch that was cut but not stapled (Figure 2B).

The lesion injury occurred at the deepest corner of vessel exposure, making thoracoscopic repair difficult. Thus, an emergent conversion to thoracotomy was performed; the A1 vessel was clamped first and then ligated with 0 silk sutures and with clips, resulting in bleeding resolution (Figure 2C). Radical lymphadenectomy completed the operation, and one drainage was left in the pleural cavity. Video S1 summarized the entire operation. Total blood loss was estimated at 350 mL. The blood pressure and hemoglobin remained stable; thus, no blood transfusion was required.

Histological examination of the resected lobe revealed T1bN0 adenocarcinoma. The postoperative course was unremarkable. The drainage was removed on the fourth postoperative day, and the patient was discharged the day after. At 5 months follow‐up, the patient was well without recurrence.

A written informed consent was obtained from the patient for the surgical procedure and for the utilization of data and pictures for scientific purposes only.

## 3 Discussion

The presence of calcified peri‐arterial lymph nodes is not a main contraindication for thoracoscopic lobectomy. However, it remains a challenging situation and one of the major causes of massive bleeding and of unplanned conversion to thoracotomy. The pulmonary arteries are fragile and require careful surgical manipulation. In this case, a calcified lymph node tenaciously adhered to the mediastinal trunk of PA, and all thoracoscopic maneuvers to dissect it tore the vessels with high risk of injury. Therefore, fear of massive bleeding led us to fire the mediastinal trunk together with the lymph node.

Previous authors [1, 2] safely performed this strategy in similar situations. Li et al. [1] fired together the target artery with a calcified lymph node after bronchus resection. Liu et al. [2] fired the bronchus, calcified lymph node, and pulmonary artery together when it was difficult to dissect the target bronchus or pulmonary artery separately. In all previous cases, no complications such as bronchopleural fistula or intraoperative bleeding were reported.

Conversely, in this case, the stapler failed to close the vessel, resulting in massive bleeding which needed an emergency conversion to thoracotomy to fix it. Some authors [4, 5] reported massive intraoperative bleeding caused by stapler malfunction due to the incorrect alignment of the anvil on manufacture. However, in our case, the trouble was not due to the defect of the machine but to the imprecise match between staple height and tissue thickness. We used a standard white vascular cartridge that probably was unable to staple a thick tissue, such as the vessel with a calcified lymph node. The thickest stapler was used in similar cases (green cartridge instead of standard vascular white cartridge) [1, 2], but it remained an off‐label use that should not be recommended. Thus, the best strategy remained to cut the pulmonary artery where the lymph nodes are not attached, and the plasty of the pulmonary artery should be considered if the lymph nodes cannot be dissected from the vessels. A vessel loop can be placed in advance around the pulmonary artery proximally, double looped around it, and gently pulled up to completely stop its blood flow in case of unexpected intraoperative bleeding [6, 7, 8]. All team members in the operating room, including anesthesia and nurses, should be ready to prepare for urgent thoracotomy if the bleeding cannot be fixed under thoracoscopy. A recent review showed that unplanned converted VATS did not increase the postoperative complications and did not negatively affect survival compared to successful thoracoscopic lobectomy and/or upfront thoracotomy lobectomy. The anesthesia team placed another intravenous line if needed; the blood was brought into the operating room and administered if the patient was hemodynamically unstable from hemorrhage, and the hemoglobin levels were less than 7 g/dL [6, 7, 8].

In conclusion, our case confirms that the calcified hilar lymph nodes are a technical challenge; if the surgeons are not confident in managing this situation under thoracoscopy, conversion to thoracotomy should never be forgotten. Open surgery could facilitate the dissection of calcified lymph nodes and safely fix unexpected bleeding due to vascular lesions.

## Author Contributions

Plannes and writes paper: A.F. Collect clinical data and images: B.L., M.M., F.C., V.D.F., and F.C. Reviews the paper: G.V.

## Conflicts of Interest

The authors declare no conflicts of interest.

## Supporting information


**Video S1.** Video edited the main steps of the procedure as the hilar structures ligation, the massive bleeding and its resolution.
